# Local Peculiarities and Diseases of the Teeth in Europe

**Published:** 1875-11

**Authors:** C. M. Wright


### LOCAL PECULIARITIES AND DISEASES OF THE TEETH IN EUROPE.

BY C. M. WRIGHT, D. D. S.

*Read before the American Dental Society of Europe.*

Mr. President and gentlemen, this and the fifth subject of discussion are to me very interesting and I think important questions. A knowledge of them may help us more in our daily fight against the enemy. It may save our muscles, our gold, our patience and our patient's feelings a great deal, and only call in a little more of the exercise of our brains in the diagnosing and prescribing departments. The more we know of the cause of disease, the better equipped are we for successfully combatting it. If a general has accurate knowledge of the motives and movements of the enemy he can lay his plans with a certaintv of success, and can smile calmly at the idea of defeat. So if we know the causes of our enemy as well as his motives and movements, we shall be successful as dentists.

Since coming to Switzerland I have been struck with the difference in the teeth, their diseases and peculiarities, from the teeth I became acquainted with in Cincinnati, Ohio; and in our discussions of last year, in experience here and the experiences given by our brothers at home through the transactions of their societies. I have felt an increased desire to have the peculiarities and diseases of teeth of different nations and sections of the country discussed and explained. Hoping to hear from other members I will give the result of my observations in Basel, on the Rhine. First. White (sensitive) caries abounds in children's teeth, affecting them as though from a vitiated mucous secretion about the neck of the teeth on their approximate surfaces, often under the free margin of the gum. This sort of caries is not only difficult of access, but difficult to discover. No casual glance

*will* discover it, and only the most careful *feeling* with proper shaped excavators or probes, can expose it till it has made a large and dangerous cavity. This kind of caries occurs between all the teeth from the incisors to the molars. Hence we have to look to our cervical walls with the utmost care, and wedge deeply and thoroughly in our manipulations. Second. Irregularities in second dentition abound, and where in Cincinnati it is rare, in Basel it is common to find persons of forty years of age with a temporary cuspid or two in either maxillary, remaining. I have seen a pivot tooth inserted in a temporary cuspid root, and to see them filled with gold and doing the work of their usual follower, is not at all rare.

Third. The disease, I suppose, we should call abrasion, chemical abrasion of the necks of teeth, is very very common. Persons with long, fine looking white teeth, have groves or large smooth notches, like the *blaze* on a tree, at the necks of the teeth. These places are sometimes sensitive and often, not at all, and seem to increase till the pulp is exposed. The teeth that are so affected are more frequently the cuspid and bicuspid on their labial and buccal sides, though it is found in all the teeth.

In Basel many of these notches have been filled with gold, and I have tried nitrate of silver applications in the smaller notches, I think, with some success. When the silver unites with the dentine, forming the black protection, the disease is apparently arrested. Sometimes the surface is so glassy and smooth like the enamel or a fine porcelain tooth, that the nitrate does not seem to have the desired effect, though I have tried the stick and the solution of nitrate of silver.

Fourth. It is quite common here to find the front upper teeth projecting frightfully over the lower incisors. The bicuspids and molars being too short for the incisors and the lower ones pushing like fiends at the upper, till they almost creep out of the mouth and over the under lip. How can this irregularity be treated in the most simple way?

Fifth. Why do these diseases appear here oftener than in Cincinnati? Why are the teeth of the valley of the Rhine, so generally liable to disease? I ask the American Dental

Society, of Europe, or the societies at home for an answer. Can Dr. John Allen's theory of the coarse bread, the half ground wheat bread and, *vice versa*, the too fine flour, account for the difference in teeth? Are the Scotch and English teeth better with their glassy enamels, because oatmeal and such grains are used more? Has *climate*, *water*, *air* anything to do with the peculiarities and diseases of the teeth? Men's teeth here as elsewhere, are better as a rule, than women's, among the aristocrats and peasants, and the peasant woman lives as a man. Let us have reasons, sound and good my brethren, if possible.

